# Calculating Thermodynamic Factors for Diffusion Using the Continuous Fractional Component Monte Carlo Method

**DOI:** 10.1021/acs.jctc.3c01144

**Published:** 2023-12-19

**Authors:** Thejas Hulikal Chakrapani, Hadi Hajibeygi, Othonas A. Moultos, Thijs J. H. Vlugt

**Affiliations:** † Reservoir Engineering, Geoscience and Engineering Department, Faculty of Civil Engineering and Geosciences, Delft University of Technology, 2628 CN Delft, The Netherlands; ‡ Engineering Thermodynamics, Process and Energy Department, Faculty of Mechanical, Maritime and Materials Engineering, Delft University of Technology, 2628 CB Delft, The Netherlands

## Abstract

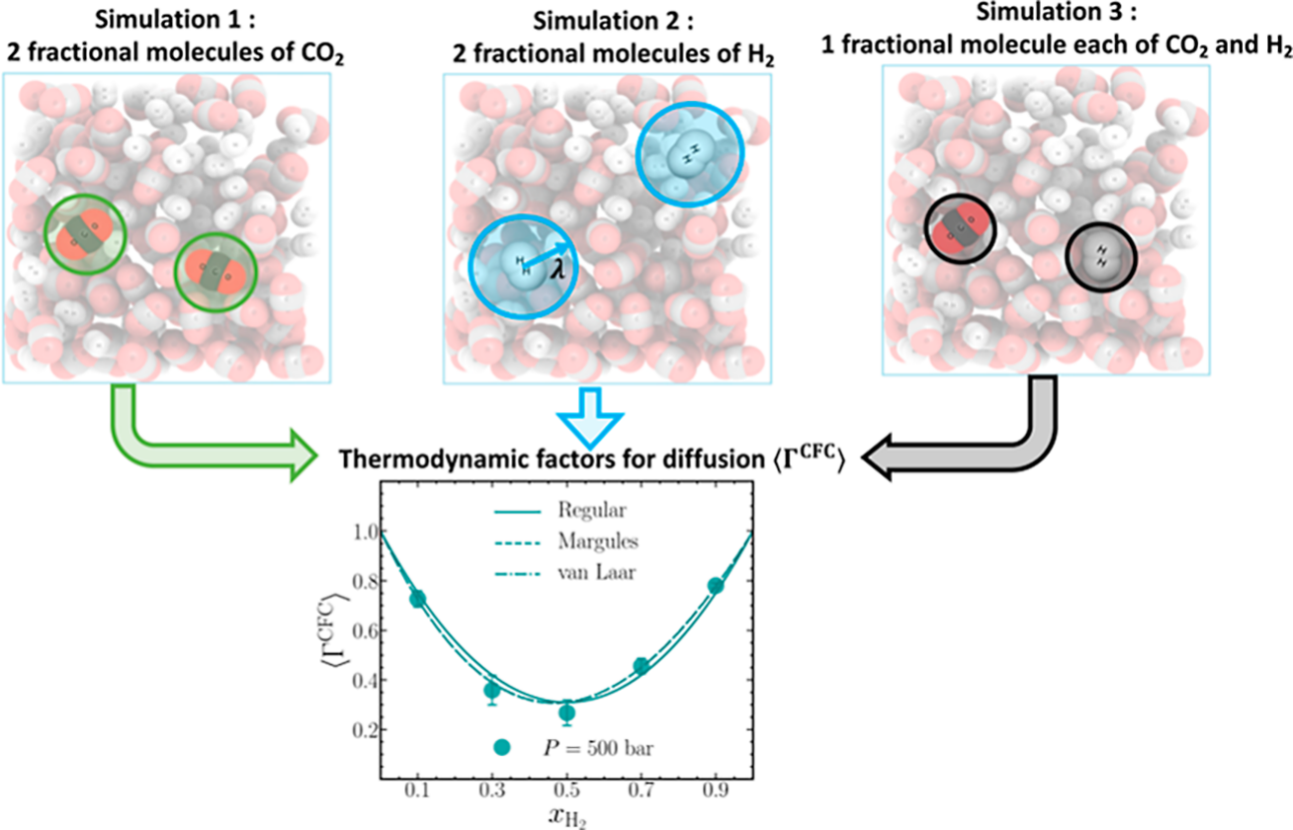

Thermodynamic factors for diffusion connect the Fick and Maxwell–Stefan diffusion coefficients used to quantify mass transfer. Activity coefficient models or equations of state can be fitted to experimental or simulation data, from which thermodynamic factors can be obtained by differentiation. The accuracy of thermodynamic factors determined using indirect routes is dictated by the specific choice of an activity coefficient model or an equation of state. The Permuted Widom’s Test Particle Insertion (PWTPI) method developed by Balaji et al. enables direct determination of thermodynamic factors in binary and multicomponent systems. For highly dense systems, for example, typical liquids, it is well known that molecular test insertion methods fail. In this article, we use the Continuous Fractional Component Monte Carlo (CFCMC) method to directly calculate thermodynamic factors by adopting the PWTPI method. The CFCMC method uses fractional molecules whose interactions with their surrounding molecules are modulated by a coupling parameter. Even in highly dense systems, the CFCMC method efficiently handles molecule insertions and removals, overcoming the limitations of the PWTPI method. We show excellent agreement between the results of the PWTPI and CFCMC methods for the calculation of thermodynamic factors in binary systems of Lennard-Jones molecules and ternary systems of Weeks–Chandler–Andersen molecules. The CFCMC method applied to calculate the thermodynamic factors of realistic molecular systems consisting of binary mixtures of carbon dioxide and hydrogen agrees well with the NIST REFPROP database. Our study highlights the effectiveness of the CFCMC method in determining thermodynamic factors for diffusion, even in densely packed systems, using relatively small numbers of molecules.

## 1 Introduction

Multicomponent mass transfer by diffusion is crucial for the design and optimization of many industrial processes.^1,2^ Self- and mutual diffusion are the two main categories. Self-diffusion is reflected by the displacements of individual molecules,^3^ while mutual diffusion describes the collective molecular transport of a species due to a concentration or chemical potential gradient. Mutual diffusion in gases and liquids often dictates the design principles of chemical reactors and separators,^4,5^ and is traditionally treated using the Fick and Maxwell–Stefan (MS) approaches.^6^ For an *n*-component system, Fick’s approach linearly correlates the mass flux of a component to the concentration gradients of all *n* species in a mixture. The proportionality constants are identified as the Fick diffusion coefficients. There are (*n* – 1)^2^ independent Fick diffusion coefficients.^5^ The MS approach uses nonequilibrium thermodynamics to relate the drag force experienced by a species to its chemical potential gradient, considering interactions with all other species.^5^ The chemical potential gradients act as driving forces for diffusion, so the Maxwell–Stefan diffusion coefficient emerges as an effective inverse friction coefficient that balances this driving force.^2^ As chemical potential gradients are not directly accessible, one needs to convert them into concentration gradients, making Fick coefficients the preferred choice for experiments.^2,5,7^ In a molar reference frame, the diffusion coefficients from the Fick and MS frameworks can be related via the so-called thermodynamic factors for diffusion,^7^ as follows
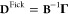
1where **D**^**Fick**^ is a square matrix of size (*n* – 1) consisting of the Fick diffusivities, **B** is a square matrix of the same size, which depends on the *n*(*n* – 1)/2 MS diffusivities,^5^ and the matrix **Γ** contains the thermodynamic factors for diffusion. The elements of **Γ** are given by^7^
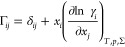
2where *T* is the absolute temperature, and *p* is the pressure. The term Σ enforces that during differentiation the mole fractions {*x*_*i*_} of all species remain constant, except for the *n*th component. This constraint ensures that the mole fractions sum to unity during the differentiation. δ_*ij*_ is the Kronecker delta, equal to 1 when *i* equals *j* and 0 otherwise, and Γ_*i*_ is the activity coefficient^7^ of component *i*. Note that for an *n*-component system, the **Γ** matrix containing (*n* – 1)^2^ elements is not symmetric.^5,7^ For an ideal *n*-component mixture, we have Γ_*i*_ = 1, so Γ_*ij*_ = δ_*ij*_.

Thermodynamic factors **Γ** for binary and multicomponent systems can be determined from experiments and molecular simulations.^7^ Activity coefficient models or equations of state can be fitted to experimental or simulation data, from which the elements of **Γ** can be obtained by differentiating eq 2.^7^ In molecular simulations, activity coefficients can be obtained from Widom’s Test Particle Insertion (WTPI) method.^8−10^ This indirect approach involves differentiating a fitted model for γ_*i*_, and the quality of the obtained thermodynamic factors is dependent on the accuracy of the activity coefficient model. The variability of the elements of **Γ** with different activity coefficient models further complicates their determination. In molecular simulations, besides fitting the simulation data to activity models or equations of state, there are alternative approaches to obtain thermodynamic factors for diffusion: (1) Kirkwood-Buff Integrals (KBIs);^11−19^ (2) simulations in the grand-canonical ensemble;^20^ and (3) the Permuted Widom Test Particle Insertion (PWTPI) method.^21,22^ The Kirkwood–Buff method^18^ relies on the evaluation of radial distribution functions and their integration over volume. This avoids pitfalls associated with molecular insertions at high densities, as in the grand-canonical ensemble and Widom’s test particle insertion method,^10^ and it provides a method to obtain thermodynamic factors directly, i.e., without fitting the data to an activity coefficient model or an equation of state. KBIs only converge for large systems and necessitate a nontrivial interpretation,^12−14^ thus, can be cumbersome to compute. For systems with *n* > 2, the expressions for obtaining Γ_*ij*_ from KBIs are complex.^23,24^ Around a decade ago, to obtain thermodynamic factors directly from molecular simulations, the PWTPI method^21,22^ was introduced as a modified version of the conventional WTPI method.^8,10^ In this method, combinations of independently generated test molecules for a single system state are used to directly compute the composition derivative of the excess chemical potential of the system and thereby the thermodynamic factors for diffusion. This method avoids explicit differentiation of excess chemical potentials or activity coefficients and provides a direct route to calculate thermodynamic factors from a single simulation, at roughly the same computational cost as the WTPI method. The PWTPI method faces the same challenges at high densities as simulations in the grand-canonical ensemble and the WTPI method, so it is unsuitable for systems with liquid-like densities at standard conditions.^10^ WTPI and PWTPI can be classified as single-step insertion methods, where whole test molecules are inserted in a single Monte Carlo step. The Configurational Bias Monte Carlo (CBMC) method is an example of a single-step insertion method commonly used to insert fragments of large molecules like polymers.^10^ For successful test molecule insertions in the WTPI, PWTPI, and CBMC methods, cavities/voids must be available within the simulation box in which the test molecule can fit. In dilute systems, voids are plentiful, while the availability of cavities in dense systems is exceedingly rare, posing a challenge for successful test molecular insertions. Should a void be present and successful insertion of a test molecule occur in rare instances, these infrequent events significantly impact the statistics, leading to imprecise estimations of free energies.^10^ For example, Torres-Knoop et al.^25^ compared the CBMC and the CFCMC methods for computing adsorption properties in porous materials like zeolites and metal–organic frameworks. The authors showed that the efficiency of insertion depends on the density of the system and that the CBMC method can yield unphysical results when systems are dense. To summarize, Monte Carlo methods such as the WTPI, PWTPI, and CBMC that attempt to insert molecules in a single step fail when molecular systems possess liquid-like densities.^10^

There is, thus, a clear need to develop an efficient method to calculate thermodynamic factors for diffusion in dense systems that is also computationally inexpensive. In this work, we present a modified version of the PWTPI method to directly calculate thermodynamic factors for diffusion Γ_*ij*_ from molecular simulations that overcomes the limitations at high densities.^26−28^ The Continuous Fractional Component Monte Carlo (CFCMC) method^26−31^ is used to facilitate molecular test insertions at high densities, thereby overcoming the limitation of the PWTPI method. The CFCMC method uses the gradual insertion and removal of so-called fractional molecules by coupling their interactions to the surrounding molecules by the parameter λ. By setting up an expanded ensemble in which λ is an additional degree of freedom, the effect is that molecules can be inserted or removed in multiple stages or Monte Carlo trial moves so that the surrounding molecules can adapt to the insertion or removal of test molecules. The CFCMC method has been successfully used in the grand-canonical,^28^ Gibbs,^29^ osmotic,^28^ and reaction^32^ ensembles. It has also been used for calculating the free energies^28,30,33,34^ and partial molar properties of fluids.^35^ We first establish equivalence with the PWTPI method for a binary system of molecules interacting via Lennard-Jones (LJ) potentials and a ternary system of repulsive molecules interacting through the Weeks–Chandler–Andersen (WCA) potential.^10,36^ Thermodynamic factors for diffusion are computed for molecular systems with realistic force fields, namely, binary mixtures of hydrogen and carbon dioxide. These binary mixtures are pertinent for hydrogen storage in large porous reservoirs, and their thermodynamic factors for diffusion have not been calculated using molecular simulations.^37−39^ In subsurface hydrogen storage, a highly compressible gas like supercritical carbon dioxide is also injected to confer mechanical stability to the reservoir.^40,41^ These gases form nonideal binary mixtures due to carbon dioxide’s supercritical behavior, and predicting their mutual diffusion requires thermodynamic factors for diffusion. Based on relevance to subsurface hydrogen storage applications, thermodynamic factors for diffusion at 5 mol fractions are calculated at pressures of 50 and 500 bar and a temperature of 323.15 K. Thermodynamic factors for diffusion are then compared to three activity coefficient models fitted to the Gibbs-excess energy data obtained from the NIST Reference Fluid Thermodynamic and Transport Properties (REFPROP) database.^42^ We demonstrate that our method can accurately predict thermodynamic factors using systems consisting of about 100 molecules, provided that finite-system size effects are eliminated.

The article is organized as follows. In Section 2, the expressions from the PWTPI and the CFCMC methods for the thermodynamic factors of binary and ternary systems are presented. The simulation details and force field used in this work are described in Section 3. In Section 4, the thermodynamic factors for diffusion computed for the binary and ternary systems from CFCMC are compared to the values from the PWTPI method. The values of the thermodynamic factors for diffusion for binary mixtures of hydrogen and carbon dioxide are presented and discussed. Our findings are summarized in Section 5.

## 2 Theory

Mathematical expressions for the elements of the Γ^PWT^ matrix from the PWTPI method^21,22^ for binary and ternary systems are presented and briefly discussed in this section. Equivalent expressions for the CFCMC method are derived. For a detailed background on these topics, the reader is referred to the original articles on the PWTPI method^21,22^ and the CFCMC simulation technique.^26−28,31^

### 2.1 Thermodynamic Factors from the Permuted Widom’s Test Particle Insertion Method

In an *n*-component system, the relation of the activity coefficient Γ_*i*_ of the *i*th component (see eq 2) to its chemical potential μ_*i*_ is^9^
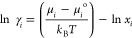
3where μ_*i*_^o^ is the chemical potential of the pure component *i*, and *x*_*i*_ is the mole fraction of component *i*. The chemical potential μ_*i*_ of a component *i* in a multicomponent system is defined as the change in the Gibbs free energy *G* of the system upon the addition of a single molecule while fixing the composition of the remaining *n* – 1 components at constant temperature and pressure
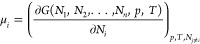
4
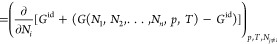
5where *N*_*j*_ is the total number of molecules of the *j*th component, *G*^id^ is the ideal gas contribution to the Gibbs free energy, *p* is the pressure, and *T* is the absolute temperature. Adding and subtracting *G*^id^ in eq 5 eliminates finite-size effects^22^ as the contribution of *G*^id^ to values in the **Γ** matrix is known a priori. Upon inspection of eqs 2–5, it is evident that the values of the **Γ** matrix are second-order derivatives of the Gibbs free energy with respect to the number of molecules. It is often difficult to analytically express the Gibbs free energy in terms of thermodynamic variables like pressure, temperature, and the number of molecules of the components due to the complexity of the system. In the PWTPI method, the second-order derivatives of the Gibbs free energy are expressed as two successive first-order forward differences.^21^ Following Balaji et al.,^22^ the elements of the thermodynamic factors estimated using the PWTPI method Γ^PWT^ can be derived by combining equations eqs 2–5. The diagonal and off-diagonal elements of the Γ^PWT^ matrix for an *n*-component system equal
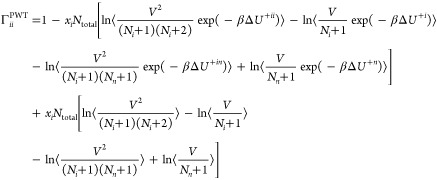
6
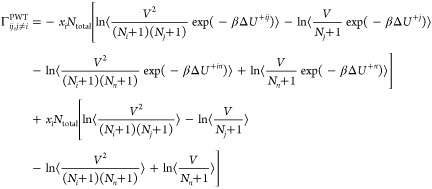
7where  is the number of molecules in the system, *V* is the volume of the simulation box, Δ*U*^+*i*^ and Δ*U*^+*ij*^ are the changes in the potential energy of the system due to the addition of a single molecule of type *i* and two molecules of types *i* and *j* (where *j* can equal *i*), respectively. , where *k*_B_ is the Boltzmann constant. The terms enclosed within parentheses ⟨···⟩ denote ensemble averages at constant temperature and pressure. Note that the term Δ*U*^+*in*^ emerges from the requirement that during differentiation of eq 2, the mole fractions of all components be held constant except the *n*th.^7,21^ The thermodynamic factors are influenced by the system size due to the extensive nature of *N*_*i*_, *N*_*j*_, and *V*, as pointed out by Balaji et al.^21^ in their original manuscript on the PWTPI technique. To remove this finite-size dependence, we invoke the thermodynamic limit, where *N*_total_ → ∞ while keeping all ratios *N*_*i*_/*N*_*j*≠*i*_ fixed and scaling *V* proportionally with *N*_total_ to maintain a constant density, ρ = *N*_total_/*V*. In this limit, terms like (*N*_*i*_ + 1)/*V* converge to *N*_*i*_/*V* = ρ_*i*_, an intensive thermodynamic property, effectively eliminating finite system effects. In a subsequent work, Balaji et al.^22^ removed these finite-size effects by subtracting the ideal gas contribution to the total Gibbs energy, as shown in eq 5.

The matrix of thermodynamic factors for a binary system consists of a single element and can be obtained by setting *n* = 2 and *i* = 1 in eq 6
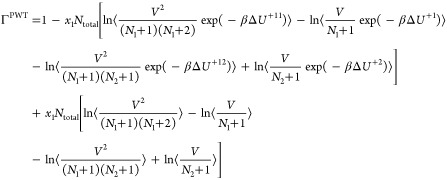
8

An alternate expression can be derived by setting *n* = 2 and *i* = 2 in eq 6, which yields an identical average value for Γ^PWT^.^21^

The matrix of thermodynamic factors Γ^PWT^ for a ternary system follows by substituting *n* = 3 into eq 7(21,22)
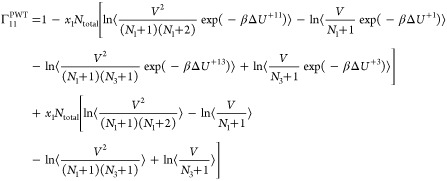
9
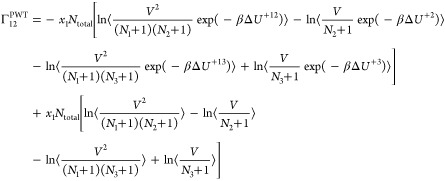
10
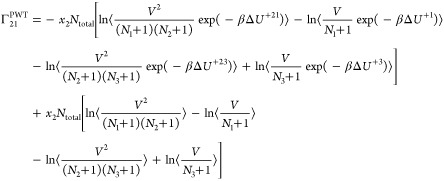
11
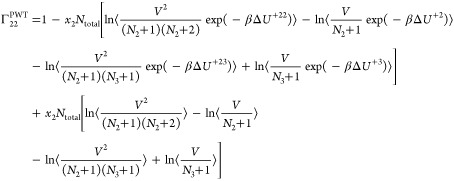
12

For a mixture of ideal gases (Δ*U*^+*ij*^ = 0), eqs 9–12 yield an identity matrix.^21^ In the case of a ternary color mixture, where the interaction energies are independent of the molecule types (Δ*U*^+*i*^ and Δ*U*^+*ij*^ are identical for all *i* and *j*), eqs 9–12 again yield an identity matrix.^21^

### 2.2 Thermodynamic Factors from the Continuous Fractional Component Monte Carlo Method

The CFCMC method aids in the gradual insertion and deletion of test molecule(s) by coupling their interactions to the surrounding molecules by a parameter λ. The parameter λ is an additional degree of freedom in an expanded ensemble. Groups containing multiple fractional molecule(s) can be defined in the simulation.^28,30^ At λ = 0, fractional molecules behave like ideal gas molecules with no interactions with the surrounding molecules, while at λ = 1, fractional molecules develop full interactions with the surrounding molecules.^26−28^

A biasing potential *W*(λ) is added to facilitate sampling of λ values.^28,30^ For a detailed background on CFCMC, the reader is referred to the original articles.^26−28,30^

Poursaeidesfahani et al.^29^ derived simple relations equating the ensemble averages calculated in the conventional Gibbs Ensemble and the CFC version of the Gibbs Ensemble. To define thermodynamic factors within the CFCMC framework (**Γ**^CFC^), we need to find expressions equivalent to the individual terms appearing in eqs 6 and 7, corresponding to the PWTPI method. Following Poursaeidesfahani et al.,^29^ the ensemble averages in the CFCNPT and PWTPI frameworks can be related as^29^
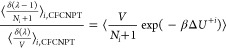
13
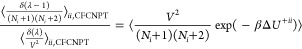
14
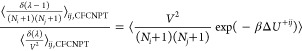
15
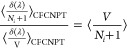
16
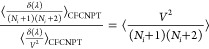
17
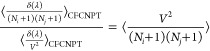
18where the bracket ⟨···⟩_*i*/*ii*/*ij*,CFCNPT_ denotes the ensemble average at constant pressure and temperature and in the presence of fractional group(s). Fractional groups of two fractional molecules of type *i* (*ii*), one fractional molecule of type *i* and one fractional molecule of type *j* (*ij*), or a single fractional molecule of type *i* (or *j*) correspond to independent simulations. At λ = 0, the molecules within the fractional group exhibit no interactions with the surrounding molecules, transitioning into whole molecules with complete interactions at λ = 1. The terms on the left side of eqs 16–18 can be computed using any fractional group since they all yield (statistically) identical values when λ = 0. The function δ(λ) is the Dirac delta function. In the above discussion, system states are sampled in the presence of a biasing potential *W*(λ),^29^ which ensures uniform sampling of λ. To calculate the ensemble average of the type ⟨*X*⟩_*i*/*ii*/*ij*,CFCNPT_, appropriate weights are multiplied to *X* to obtain the Boltzmann averages.^29^ The thermodynamic factors **Γ**^CFC^ for an *n*-component system in the CFCNPT ensemble can then be readily obtained by replacing the individual terms in eqs 6 and 7 using relations from eqs 13–18. The thermodynamic factor Γ^CFC^ in the CFCNPT method for a binary system reads as
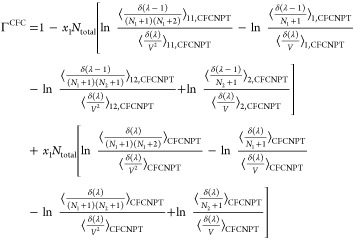
19

This expression is derived in the presence of a fractional molecule of type 1. An equivalent expression for Γ^CFC^ derived using a fractional molecule of type 2 where *n* = 1 is
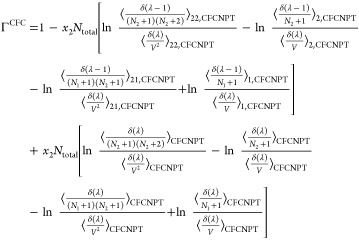
20

The expressions for the **Γ**^CFC^ matrix for a ternary system are
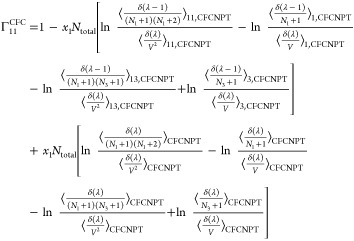
21
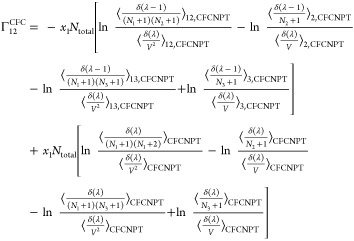
22
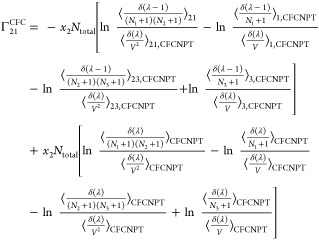
23
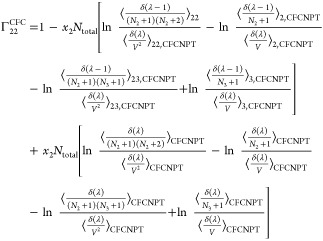
24

### 2.3 Using Gibbs–Duhem Equations for Simplifying Thermodynamic Factors

The generalized Gibbs–Duhem relation at constant temperature and pressure constrains the changes in the partial molar property of a multicomponent mixture.^43,44^ This was used by Balaji et al.^21,22^ to achieve better statistics and faster convergence of the elements of the **Γ**^CFC^ matrix. Using the Gibbs–Duhem equations for a binary system yields^21,22^
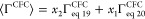
25which on expansion reads
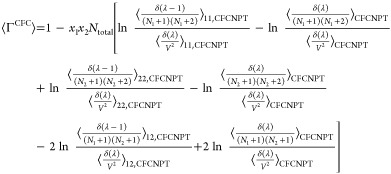
26where the common term *x*_1_*x*_2_*N*_total_ has been factored out to simplify the expression. The application of the Gibbs–Duhem equation eliminates terms emanating from single-molecule test insertions; see eq 26. ⟨Γ^CFC^⟩ converges faster toward the thermodynamic factor than eqs 19 or 20.^21,22^ Invoking the Gibbs–Duhem relations for a ternary system yields^21,22^
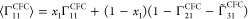
27
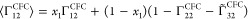
28
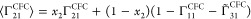
29
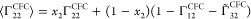
30where  and  are defined as
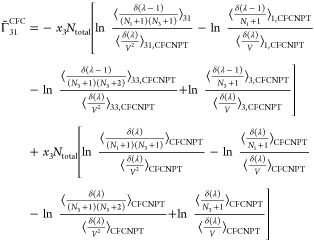
31
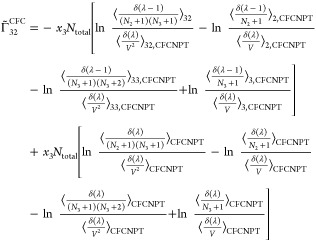
32

Note that the notation ∼ on  and  emphasizes that these elements do not belong to the matrix of thermodynamic factors **Γ**^CFC^ of a ternary system since Γ_*ij*_^CFC^ is only defined for *i* ≤ 2 and *j* ≤ 2.

The number of simulations and the associated terms required to compute thermodynamic factors for binary and ternary systems are summarized and tabulated in Tables 1 and 2. For a binary system, application of the Gibbs–Duhem equation (eq 25) results in the elimination of single molecule test insertion terms.^21,22^ The single-molecule test insertion terms for a ternary system do not cancel, barring exceptions, e.g., when *x*_1_ = 0.5, *x*_2_ = 0.25, and *x*_3_ = 0.25.^21,22^ For binary and ternary systems, terms involving molecules *i* and *j* result in identical ensemble averages as terms *j* and *i*. The binary and ternary systems require three and nine independent simulations, respectively, at a given composition of the system.

**Table 1:** List of Independent CFCMC Simulations Required to Compute the Thermodynamic Factor Γ^CFC^ at a Given System Composition for Binary Systems, See Eq 26^a^

simulation	molecule(s) in fractional group	term
1	1,1	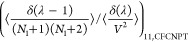
2	1,2	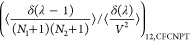
3	2,2	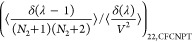

a Each row lists the simulation number, the molecule types in the fractional groups, and the associated term in eq 26. Inserting a fractional group consisting of molecules 1 and 2 results in the same ensemble average as when the order is reversed.

**Table 2:** List of Independent CFCMC Simulations Required to Compute the Matrix of Thermodynamic Factors Γ^CFC^ at a Given System Composition for Ternary Systems, See Eqs 21–24 and Eqs 27–32^a^

Simulation	Molecule(s) in fractional group	Term
1	1	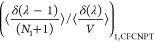
2	2	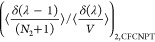
3	3	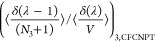
4	1,1	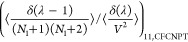
5	1,2	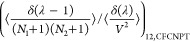
6	1,3	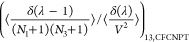
7	2,2	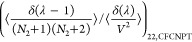
8	2,3	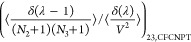
9	3,3	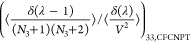

a Each row lists the simulation number, the molecule type(s) in the fractional groups, and the associated term in eqs 21–24 and eqs 27–32. Inserting a fractional group consisting of molecules *i* and *j* results in the same ensemble average as when their order is reversed.

## 3 Methods

All simulations are performed in the CFC version of the *NPT* ensemble as implemented in the open-source software Brick-CFCMC.^28,30^ Binary systems consisting of 200 molecules interacting via the Lennard-Jones (LJ) potential are simulated in a 3D simulation box with periodic boundary conditions. The interaction parameters in reduced units are σ_11_ = 1.0, σ_22_ = 1.2, σ_12_ = σ_21_ = 1.1, ε_1_ = 1.0, ε_2_ = 0.5, and ε_12_ = ε_21_ = 0.1. The LJ potential is truncated and shifted for interactions beyond 2.5σ_11_. Following Balaji et al.,^20^ simulations are performed at a reduced pressure *p* = 2.8 and temperature *T* = 2 at 9 different compositions from *x*_1_ = 0.1 to *x*_1_ = 0.9.

Ternary systems consist of 100 molecules and interact through the soft repulsive WCA potential^10,36^ in a three-dimensional box with periodic boundary conditions. The interaction parameters in reduced units are σ_*ij*_ = 1 (for all *i* and *j*), ε_*ij*_ = 1 (for all *i* = *j*), ε_12_ = 0.4, ε_13_ = 0.2, and ε_23_ = 0.5. The potential is truncated and shifted at a cutoff radius of 2^1/6^σ_11_. Following Balaji et al.,^22^ all simulations are performed at a reduced pressure of 6.8, a reduced temperature of 2, and system compositions varying between *x*_1_ = 0.1 to 0.9, while *x*_2_ = *x*_3_.

Binary mixtures of hydrogen and carbon dioxide containing 200 molecules are simulated using rigid molecular models in a three-dimensional box with periodic boundary conditions. The hydrogen molecule exhibits anisotropy due to its two nuclei and nonspherical charge distribution.^45,46^ Molecular models of hydrogen, aimed at capturing its thermodynamical behavior, can be classified as single-site^45,47,48^ or multisite models.^49,50^ Single-site models consist of a single LJ interaction site, while multisite models also include a point quadrupole to model anisotropic interactions. The three-site Marx model^45,50^ was selected for its accuracy in reproducing the bulk densities and fugacities of hydrogen at pressures up to 1000 bar.^33,51^ Quantum effects emanating at low temperatures are insignificant for the temperatures considered in this work (323.15 K) .^52,53^ Carbon dioxide is simulated as a rigid linear molecule using the TraPPE force field.^54,55^ The TraPPE force field compares favorably with the experimental data for the vapor–liquid equilibrium of pure carbon dioxide and its multicomponent mixtures for a wide range of temperatures, pressures, and compositions.^54,55^ The model has three LJ sites to model the repulsive and dispersion interactions.^54,55^ Each LJ site is also conferred a partial charge to capture electrostatic interactions. Simulations of binary systems of carbon dioxide and hydrogen are conducted at pressures of 50 and 500 bar, respectively. A fixed temperature of 323.15 K is chosen while exploring compositions from *x*_H_2__ = 0.1 to 0.9, where *x*_H_2__ is the mole fraction of hydrogen. A cutoff radius of 10 Å is used for all LJ interactions, and analytic tail corrections are applied. The interaction parameters between unlike LJ sites are defined using the Lorentz–Berthelot mixing rules.^10,56^ Electrostatic energies are computed using the Ewald summation.^57^ Cutoff distances for real-space electrostatic interactions are chosen to limit the number of *k*-vectors in Fourier space to a maximum of *k* = 8, thereby making simulations computationally less expensive.^28,57^ We choose a real-space cutoff of 11 Å with a damping parameter of α = 0.3 Å^–1^ for 50 bar. At 500 bar, we choose a real space cutoff of 19 Å with a damping parameter of α = 0.17 Å^–1^. These settings ensure that the electrostatic energies are computed with a relative precision of 10^–6^. The force field parameters for hydrogen and carbon dioxide are listed in Table 3.

**Table 3:** Interaction Parameters for the TraPPE Force Field of Carbon Dioxide, the O=C=O, and the Three-Site Marx Model for Hydrogen^a^

atom	ε/*k*_B_/[K]	σ/[Å]	*q*/[e]
O=[C]=O	27.0	2.80	0.70
[O]=C=O	79.0	3.05	0.35
H–[L]–H	3.05	2.958	–0.936
[H]–L–H			0.468

a Each row contains the LJ and the electrostatic interaction parameters for the atom highlighted between brackets []. Parameters between dissimilar species are calculated using the Lorentz–Berthelot mixing rules.^10,56^ The bond lengths O=(C=O) and H–(L–H) equal 1.16 and 0.37 Å, respectively, where the brackets () represent the bond.

Every cycle of a CFCNPT simulation contains *N*_total_ Monte Carlo (MC) moves, where *N*_total_ is the total number of molecules. In the binary and ternary systems, molecule translations, volume changes, λ changes, and CFC hybrid trial moves^28,30^ are selected with probabilities of 0.5, 0.01, 0.19, and 0.3, respectively. In hydrogen carbon dioxide binary mixtures, translations, rotations, volume changes, λ changes, and CFC hybrid trial moves^28,30^ are selected with probabilities of 0.3, 0.2, 0.01, 0.19, and 0.3, respectively. The probabilities for molecule translations, rotations, λ changes, and CFC hybrid trial moves are selected to be of similar magnitudes. Modifying these probabilities does not impact the thermodynamic properties of the system. Volume changes are computationally expensive since configurational energies need recalculation based on updated intermolecular distances after rescaling the simulation box. Consequently, volume changes are typically executed with a probability of 0.01.^28,30^ The maximum displacements for molecule translations, volume changes, rotations, and λ changes are adjusted to obtain acceptance ratios of ca. 50%. *N*_init_ initialization and *N*_equil_ equilibration cycles are performed to remove molecule overlaps and develop the biasing potential *W*(λ), respectively. A production phase lasting *N*_prod_ cycles ensures a uniform distribution of observed λ values. At every mole fraction, simulations are repeated *N*_rep_ with distinct random number generator seeds to obtain better statistics for each term in Tables 1–3. The convergence of the values of **Γ**^CFC^ is achieved by adequately sampling λ = 0 and λ = 1. Note that the delta functions in the expression for the thermodynamic factors of binary mixtures (eq 26) and ternary mixtures (eqs 21–24 and eqs 27–32) are evaluated only when λ is either 0 or 1. For intermediate values of λ, the Lennard-Jones and electrostatic interactions are scaled based on the value of λ. The complete expressions for the Lennard-Jones and electrostatic interactions as a function of λ are provided by Hens et al.^28^ The values for *N*_init_, *N*_equil_, *N*_prod_, and *N*_rep_ for all systems are provided in Table 4. Error bars are computed by dividing all simulations into 5 groups and calculating their standard deviation.

**Table 4:** Number of Initialization (*N*_init_), Equilibration (*N*_equil_), and Production (*N*_prod_) Cycles in the CFCMC Simulations for the Binary, Ternary, and Hydrogen and Carbon Dioxide Mixtures^a^

system	*N*_init_	*N*_equil_	*N*_prod_	*N*_rep_
binary	10^4^	5 × 10^6^	10^8^	40
ternary	10^4^	10^7^	10^8^	40
hydrogen + carbon dioxide	10^4^	5 × 10^5^	8 × 10^6^	100

a *N*_rep_ is the number of repetitions for each simulation, aimed at improving statistics by using different random number generator seeds and averaging the results. The computational requirements, expressed in units of CPU hours, for the calculation of thermodynamic factors at a single mole fraction are approximately 14,400 for a binary mixture of LJ molecules, approximately 10,800 for a ternary mixture of WCA molecules, and approximately 92,160 for a binary mixture of hydrogen and carbon dioxide.

## 4 Results and Discussion

As a first benchmark case, we compare thermodynamic factors computed using the CFCMC and PWTPI methods^21^ for a binary mixture of LJ molecules. In the second benchmark case, thermodynamic factors for a ternary WCA molecule system are computed using the CFCMC method and compared to the PWTPI method.^22^ The CFCMC method is used next to calculate the thermodynamic factors for diffusion in a real molecular system consisting of binary mixtures of hydrogen and carbon dioxide.

### 4.1 Binary LJ Mixtures

**Figure 1:**
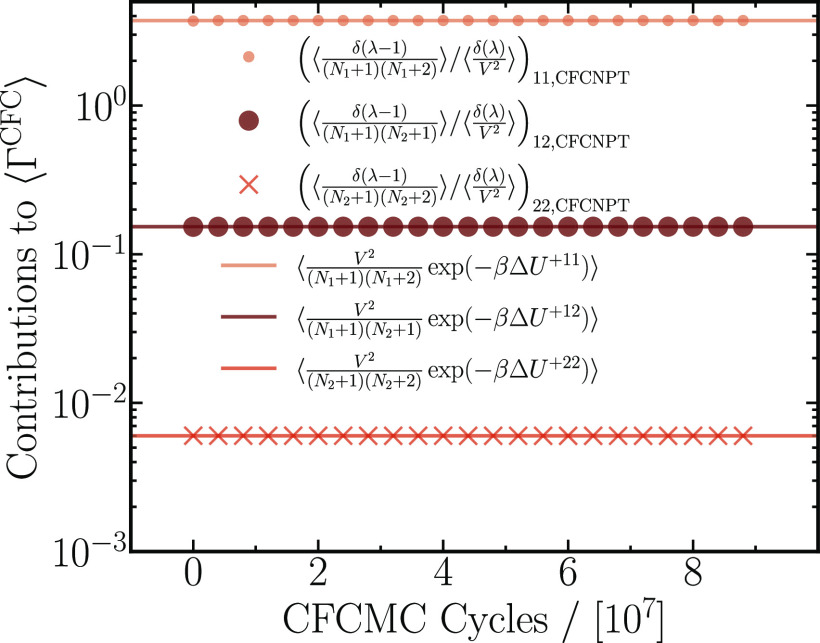
Individual terms (Table 1) from the CFCMC method, plotted as a function of the number of CFCMC production cycles for a binary mixture of LJ molecules simulated at a reduced temperature *T* = 2, reduced pressure *p* = 2.8, and *x*_1_ = 0.1. An average (reduced) density of ρ = 0.43 agrees well with the simulations from the PWTPM method.^21^ Each data point is a block average over 40 independent CFCMC simulations. Lines represent mean values from the PWTPI method (eq 8) for the corresponding CFCMC terms. Terms related to single molecule insertions are omitted as these are canceled out by the Gibbs–Duhem equation (eq 26).

For binary LJ systems, the thermodynamic factor for diffusion is calculated using eqs 19, 20, 25, and 26. Figure 1 is a numerical test of eqs 13–15, wherein the terms from the two methods show nearly exact agreement. Note that the terms in eqs 15–18, representing the single molecule insertions, are omitted since they cancel out on application of the Gibbs–Duhem equation; see eq 26. The ideal gas terms responsible for the finite-system size corrections appearing in eq 19 agree exactly with the corresponding terms from the PWTPI method (data not shown).

**Figure 2:**
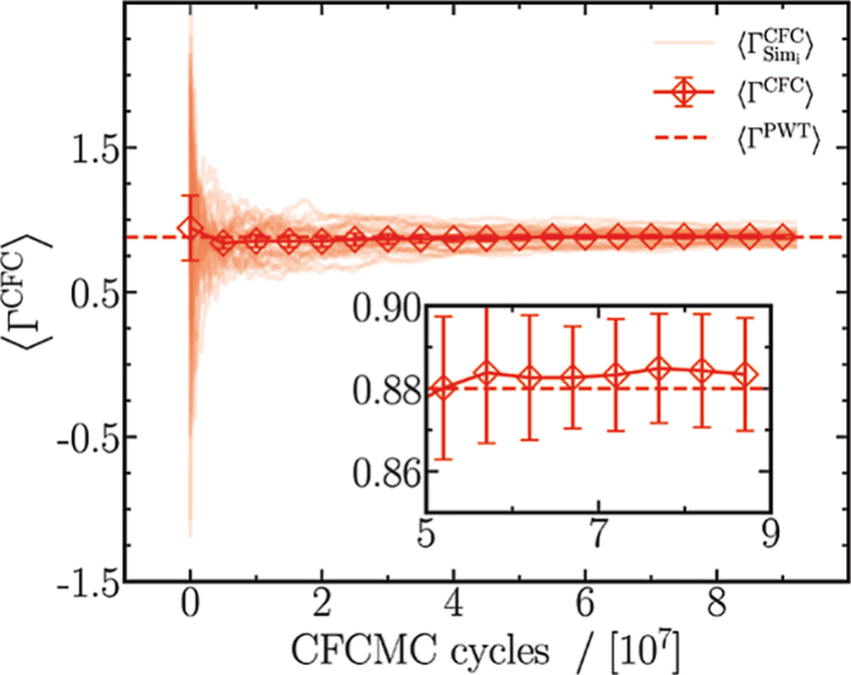
Evolution of ⟨Γ^CFC^⟩ with the number of CFCMC production cycles is plotted for a binary mixture of LJ molecules at *T* = 2 and *p* = 2.8 in reduced units and *x*_1_ = 0.1. Every symbol and the accompanying errors in measurement are computed by evaluating block-averages of  from 40 independent simulations, where *i* corresponds to the number of the independent simulations. For comparison with the PWTPI method, ⟨Γ^PWT^⟩ is plotted as a dashed line. The inset zooms in on ⟨Γ^CFC^⟩ between 5× 10^7^ and 9 × 10^7^, while retaining ⟨Γ^PWT^⟩ for comparison.

The values of ⟨Γ^CFC^⟩ obtained from applying eq 26 are shown in Figure 2 as a function of the number of CFCMC cycles. To emphasize the role of statistics, we plot  from 40 independent simulations, where Sim_i_ denotes the *i*th simulation for 1 ≤ *i* ≤ 40, while ⟨Γ^CFC^⟩ is calculated as an average of these simulations. Individual simulations show large fluctuations in the early stages of an MC simulation (10^7^ cycles) and converge toward a unique value at later stages. The fluctuations are primarily caused by infrequent sampling of the states λ = 0 and λ = 1, which vanish with the progression of the simulation. The value of ⟨Γ^CFC^⟩, in sharp contrast, achieves convergence early on with an uncertainty of approximately 2% (see inset of Figure 2). The error associated with the term ⟨Γ^CFC^⟩ can be decreased by ensuring better sampling of the λ space, achieved by either performing many simulations concurrently or running significantly longer simulations. A mean value of ⟨Γ^CFC^⟩ for the last 10^7^ cycles is calculated at every mole fraction.

**Figure 3:**
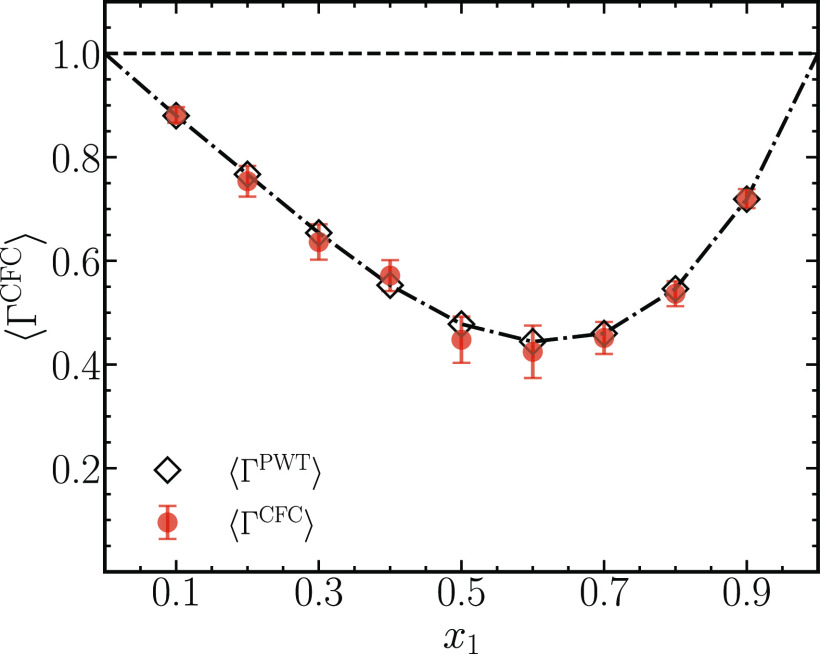
Comparison between the thermodynamic factors obtained using the CFCMC method ⟨Γ^CFC^⟩ (eq 25) and the PWTPI method ⟨Γ^PWT^⟩^21^ for a binary mixture of LJ molecules at a reduced temperature *T* = 2 and a reduced pressure of *p* = 2.8. The reduced densities range between 0.4 at *x*_1_ = 0.1 and 0.6 at *x*_1_ = 0.9. The black dashed-dotted line connecting the black diamond symbols is drawn to aid the eye.

Given the excellent agreement of the individual terms between the PWTPI and the CFCMC methods (Figure 1), we expect a similar agreement between the resulting thermodynamic factors ⟨Γ^CFC^⟩ and ⟨Γ^PWT^⟩. To facilitate comparison between the two methods, the thermodynamic factors ⟨Γ^PWT^⟩ from the original PWTPI article have been corrected for the finite-size effects.^21^Figure 3 shows that the thermodynamic factors calculated from the two methods agree within 2% for all mole fractions, except between *x*_1_ = 0.4 and *x*_1_ = 0.6, where the agreement is between 5 and 6%. Note that the thermodynamic factors from the PWTPI method Γ^PWT^ lie within the error bars of Γ^CFC^. These differences can be attributed to insufficient statistics and are expected to vanish with longer simulations. These simulations also emphasize the low computational requirements of the CFCMC method.

**Figure 4:**
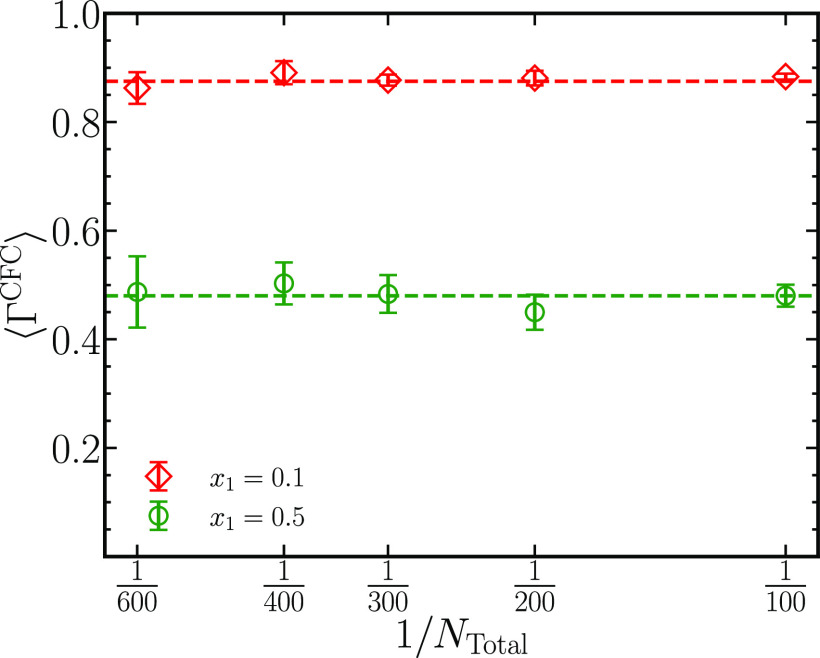
Thermodynamic factors ⟨Γ^CFC^⟩ for systems consisting of 100, 200, 300, 400, and 600 binary LJ molecules for *x*_1_ = 0.1 and *x*_1_ = 0.5. Average values of ⟨Γ^CFC^⟩ computed across all system sizes are plotted as dashed lines.

Figure 4 shows the thermodynamic factors ⟨Γ^CFC^⟩ for five system sizes *N*_total_ = 100, 200, 300, 400, and 600 at *x*_1_ = 0.1 and *x*_1_ = 0.5. The values of thermodynamic factors for diffusion at *x*_1_ = 0.1 computed from the five different system sizes yield approximately 0.88, indicating the absence of any finite-system size effects on ⟨Γ^CFC^⟩. At *x*_1_ = 0.5, the values of ⟨Γ^CFC^⟩ vary around a mean value of 0.48, with the highest uncertainty at *N*_total_ = 600. The uncertainty vanishes with longer simulations.

### 4.2 Ternary WCA Mixtures

**Figure 5:**
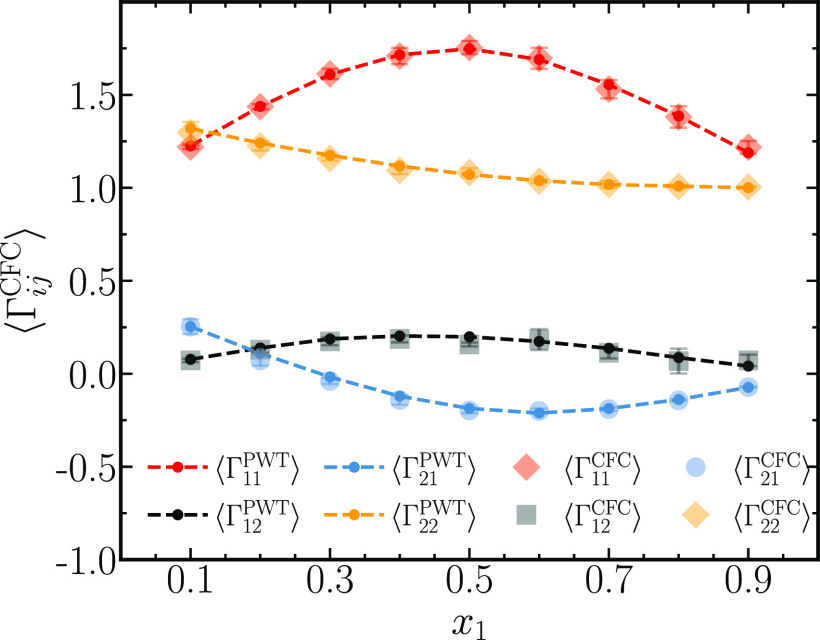
Comparison between elements of the matrix of thermodynamic factors Γ^CFC^ for ternary mixtures of WCA molecules obtained using the CFCMC method ⟨Γ_*ij*_^CFC^⟩ (eq 25) and the PWTPI method ⟨Γ_*ij*_^PWT^⟩.^22^ The simulations are performed at a reduced temperature of *T* = 2, a reduced pressure of *p* = 6.8, and at every *x*_1_, the relation *x*_2_ = *x*_3_ is satisfied. The number densities at all mole fractions were ca. 0.7 in reduced units, in agreement with Schnell et al.^20^ The dashed lines connecting the dots are drawn to aid visualization.

We now assess the performance of the CFCMC method for predicting the matrix of thermodynamic factors for diffusion Γ^CFC^ values for ternary mixtures. The densities obtained from the CFCMC simulations at all mole fractions are close to 0.7 (in reduced units) and agree with the values reported by Schnell et al.^20^ After repeating the procedure outlined for binary mixtures, the individual terms between the two methods are compared and found to be in nearly exact agreement (data not shown). ⟨Γ_*ij*_^CFC^⟩ values resulting from eqs 27–30 are shown in Figure 5. Once again, the two methods show excellent agreement for ⟨Γ_*ij*_^CFC^⟩ at all compositions of the system. Schnell et al.^20^ already showed that thermodynamic factors for ternary systems calculated using the PWTPI method are equivalent to values obtained from the grand-canonical Monte Carlo method and molecular dynamic simulations in the *NVT* ensemble, both using the KB approach. From Figure 5, it can be thus concluded that the CFCMC method provides an alternative method for calculating thermodynamic factors in ternary systems, alongside the GCMC and MD simulations. Small systems consisting of only 100 molecules are sufficient to accurately compute ⟨Γ_*ij*_^CFC^⟩.

### 4.3 Binary Mixtures of Carbon Dioxide and Hydrogen

**Figure 6:**
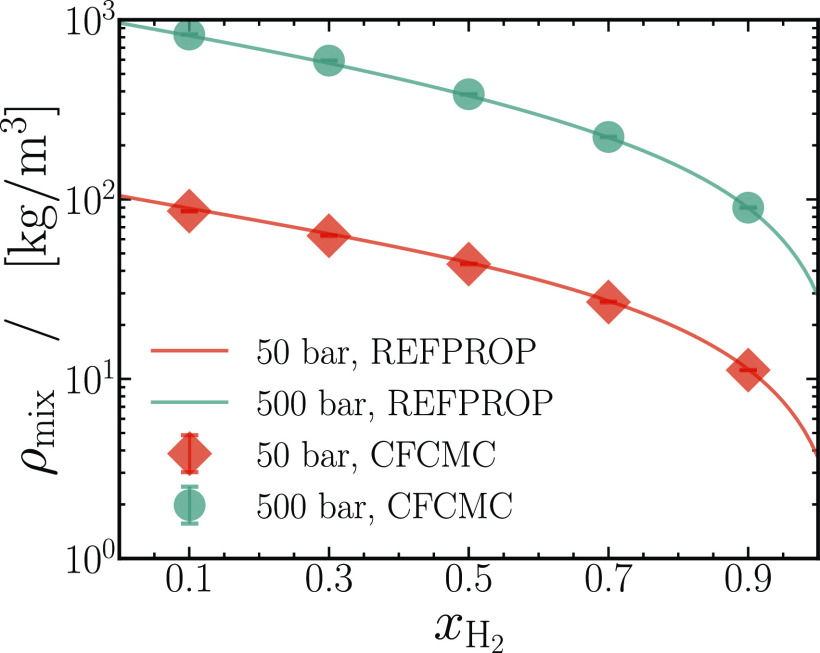
Densities of binary mixtures of carbon dioxide and hydrogen are plotted as a function of the mole fraction of hydrogen. The symbols are densities computed from CFCMC simulations, compared to the data from the NIST Reference Fluid Thermodynamic and Transport Properties (REFPROP) Database.^42^ Red symbols and lines are data at *p* = 50 bar, and green symbols correspond to *p* = 500 bar. Error bars in the simulations are smaller than the symbol sizes. All data are reported for *T* = 323.15 K. The data accompanying this plot is tabulated in Table 5.

**Figure 7:**
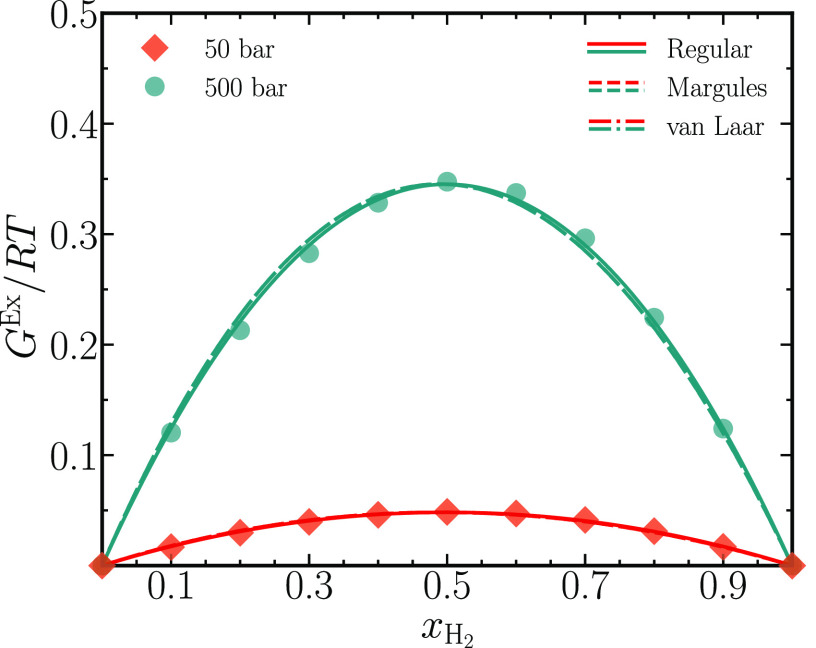
Gibbs-excess energies *G*^Ex^ in units of *RT*, where *R* is the universal gas constant, for binary mixtures of carbon dioxide and hydrogen, obtained from REFPROP^42^ are plotted as symbols for *p* = 50 and 500 bar. Gibbs-excess energies derived from three different activity coefficient models, Regular, Margules, and van Laar, are fitted to the symbols. All data are reported at *T* = 323.15 K.

**Figure 8:**
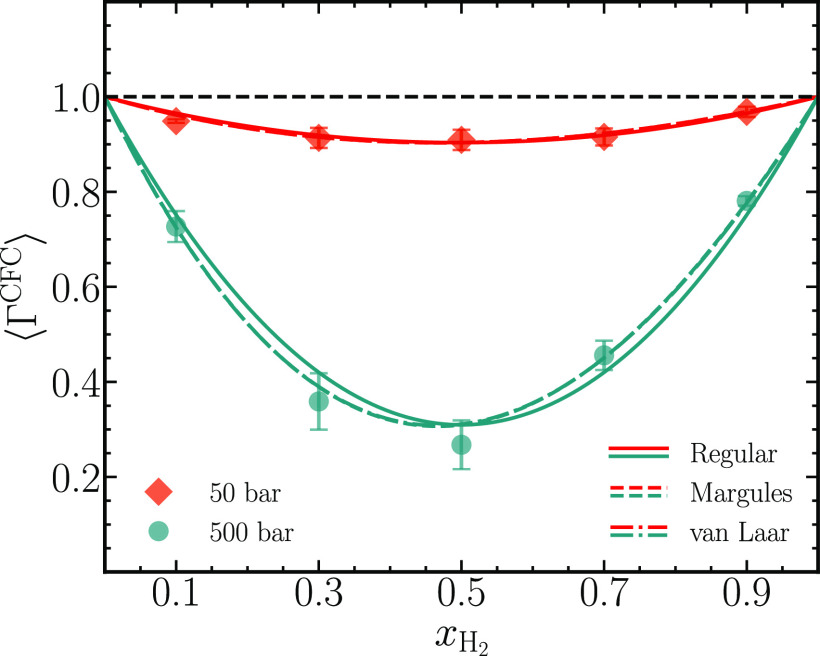
Thermodynamic factors ⟨Γ^CFC^⟩ calculated using the CFCMC method for binary mixtures of carbon dioxide and hydrogen are compared to the thermodynamic factors from differentiating the activity coefficient models in Figure 7. Simulation data at two different pressures of 50 and 500 bar and a temperature of *T* = 323.15 K are plotted using symbols. The thermodynamic factors computed from activity coefficients are shown as lines. The dashed line is used to highlight the thermodynamic factor for ideal mixtures. The data accompanying this plot are tabulated in Table 6.

We used the CFCMC method to calculate thermodynamic factors for a molecular system comprising binary mixtures of hydrogen and carbon dioxide. To the best of our knowledge, the PWTPI method has not been used previously to calculate thermodynamic factors for systems other than single LJ or WCA interaction sites. Figure 6 shows excellent agreement between the mixture densities computed from the CFCMC method and the NIST Reference Fluid Thermodynamic and Transport Properties (REFPROP) database^42^ at *p* = 50 and 500 bar. This agreement spans over two decades on the vertical axis, from exhibiting ideal gaslike behavior at 50 bar, to demonstrating liquid-like behavior at 500 bar. At *p* = 500 bar, mixtures containing minute quantities of hydrogen (*x*_H_2__ ≈ 0.1) exhibit a liquid-like behavior in which molecular insertion techniques, such as WTPI and PWTPI, will fail due to the large mixture densities (see Table 5). As a consequence, performing thermodynamic factor computations using the PWTPI method becomes impractical in such cases. Gibbs-excess energies *G*^Ex^ for the binary mixtures are extracted from REFPROP at both pressures to facilitate comparison with computed values for ⟨Γ^CFC^⟩. Figure 7 shows the Gibbs-excess energies normalized by *RT*, where *R* is the universal gas constant, plotted as a function of *x*_H_2__. Three activity coefficient models—Strict Regular, Margules, and van Laar—are fitted to the normalized Gibbs-excess energies.^7^ The strict regular model consists of a single free parameter, whereas both the Margules and van Laar models accommodate two free parameters to describe the Gibbs-excess energies. By definition, the Gibbs-excess energies approach zero for the pure systems (*x*_H_2__ = 0 and *x*_H_2__ = 1). Figure 7 shows that all three models describe the normalized Gibbs-excess energies well, barring minor differences at *p* = 500 bar. Also note that the typical energy scale for the normalized Gibbs-excess energy of ca. 0.5 (dimensionless) is typical for a dense gaseous system.^44^ The thermodynamic factors from all three models are computed from the fit coefficients by following the procedure outlined by Taylor and Kooijman.^7^Figure 8 shows a comparison between the thermodynamic factors calculated using the CFCMC method ⟨Γ^CFC^⟩ and the three activity coefficient models, and the raw data accompanying the plot are listed in Table 6. At *p* = 50 bar, good agreement is found for all three activity coefficient models, wherein the values of thermodynamic factors lie close to 1. This is in accordance with the properties of a gaseous mixture at low pressures, where it behaves like an ideal gas. For *p* = 500 bar, ⟨Γ^CFC^⟩ is significantly smaller than 1, emphasizing the nonideal behavior of the binary mixtures. It can be further inferred that the interactions between the unlike species (hydrogen–carbon dioxide) are much less favorable than the interactions of like species (hydrogen–hydrogen or carbon dioxide–carbon dioxide). On closer inspection of the figure, it is visible that the circular symbols lie closer to the Margules and van Laar models than the Strict Regular model. The Margules and van Laar model describe the simulation data accurately at *x*_H_2__ = 0.1, 0.7, and 0.9, whereas minor differences are observed at *x*_H_2__ = 0.3 and 0.5. The apparent accuracy of the Margules and van Laar models hinges on the fact that these models allow 2 free parameters, which results in better fits to the shape of Gibbs-excess energies, thus leading to a better description of the thermodynamic factors. With respect to the value of ⟨Γ^CFC^⟩, the sizes of the error bars are largest at *x*_H_2__ = 0.3 and 0.5, which we expect will diminish with more or longer simulations. Figure 8 illustrates an important point that the different activity coefficient models describing the same Gibbs-excess energies can lead to slightly different thermodynamic factors ⟨Γ^CFC^⟩. Barring the minor discrepancies between the predictions of the thermodynamic factor from the CFCMC method and the activity coefficient models, it is important to note that with a mere 200 molecules, accurate predictions can be made for the thermodynamic factors of real molecules at low and high pressures.

**Table 5:** Mixture Densities Relevant to Figure 6 for Binary Mixtures of Carbon Dioxide and Hydrogen at *p* = 50 and 500 bar and *T* = 323.15 K^a^

	*p* = 50 bar			*p* = 500 bar		
*x*_H_2__	ρ_Mix_^CFC^	ρ_Mix_^RFP^	ρ_Mix_^RFP^ [mol/m^3^]	ρ_Mix_^CFC^	ρ_Mix_^RFP^	ρ_Mix_^RFP^ [mol/m^3^]
0.1	86.1(6)	89.3	2241.9	828(4)	816.4	20,508
0.3	62.7(4)	64.4	2049.1	593(3)	573.9	18,270
0.5	43.6(2)	44.5	1932.5	385(2)	378.4	16,444
0.7	26.8(1)	27.2	1861.7	223(1)	221.2	15,138
0.9	11.2(1)	11.3	1820.7	89.9(4)	89.6	14,423

a ρ_Mix_^CFC^ and ρ_Mix_^RFP^ are the mixture densities obtained from CFCMC simulations and the REFPROP^42^ database, respectively. The corresponding molar densities expressed in units of mol/m^3^ are also tabulated. The densities are reported in units of kg/m^3^, and the uncertainty in the least significant digit of ρ_Mix_^CFC^ is provided within the brackets ().

**Table 6:** Thermodynamic Factors Relevant to Figure 8 for Binary Mixtures of Carbon Dioxide and Hydrogen at *p* = 50 and 500 bar and *T* = 323.15K^a^

	*p* = 50 bar				*p* = 500 bar			
*x*_H_2__	⟨Γ^CFC^⟩	Γ_Reg_^RFP^	Γ_Mar_^RFP^	Γ_vanLaar_^RFP^	⟨Γ^CFC^⟩	Γ_Reg_^RFP^	Γ_Mar_^RFP^	Γ_vanLaar_^RFP^
0.1	0.95(0)	0.97	0.96	0.96	0.73(3)	0.75	0.73	0.73
0.3	0.91(2)	0.92	0.91	0.92	0.36(6)	0.42	0.39	0.39
0.5	0.91(2)	0.90	0.90	0.90	0.27(5)	0.31	0.31	0.31
0.7	0.92(2)	0.92	0.92	0.92	0.46(3)	0.42	0.45	0.45
0.9	0.97(1)	0.97	0.97	0.97	0.78(1)	0.75	0.78	0.78

a ⟨Γ^CFC^⟩ represent thermodynamic factors computed from CFCMC simulations using eq 26. Γ_Mar_^RFP^, Γ_Reg_^RFP^, and Γ_vanLaar_^RFP^ are determined by fitting the Strict Regular, Margules and van Laar activity coefficient models^7^ to the Gibbs excess energies (REFPROP^42^) of the mixtures and then numerically differentiating the model with respect to *x*_H_2__. Uncertainties in the least significant digit of ⟨Γ^CFC^⟩ are provided within the brackets ().

**Figure 9:**
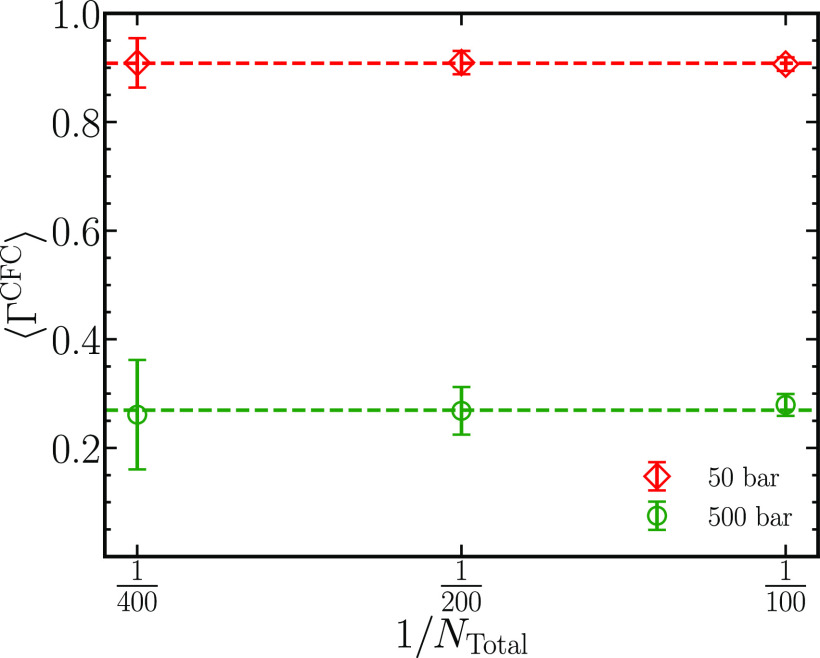
Thermodynamic factors ⟨Γ^CFC^⟩ of equimolar mixtures comprising carbon dioxide and hydrogen were computed for systems containing 100, 200, and 400 molecules. Calculations are performed at *p* = 50 and 500 bar at a fixed temperature of 323.15 K and *x*_H_2__ = 0.5. Average values of ⟨Γ^CFC^⟩ computed across all system sizes are plotted as dashed lines.

Figure 9 shows the thermodynamic factors ⟨Γ^CFC^⟩ for three system sizes *N*_total_ = 100, 200, and 400 at *p* = 50 and 500 bar and *x*_H_2__ = 0.5. The values of thermodynamic factors for diffusion at *p* = 50 bar computed from the three different system sizes yield approximately 0.90, indicating the absence of any finite-system size effects on ⟨Γ^CFC^⟩. At *p* = 500 bar, the values of ⟨Γ^CFC^⟩ vary around a mean value of 0.28, with the highest uncertainty at *N*_total_ = 400. The uncertainty will vanish with longer simulations.

### 4.4 PWTPI vs CFCMC for Dense Systems

**Figure 10:**
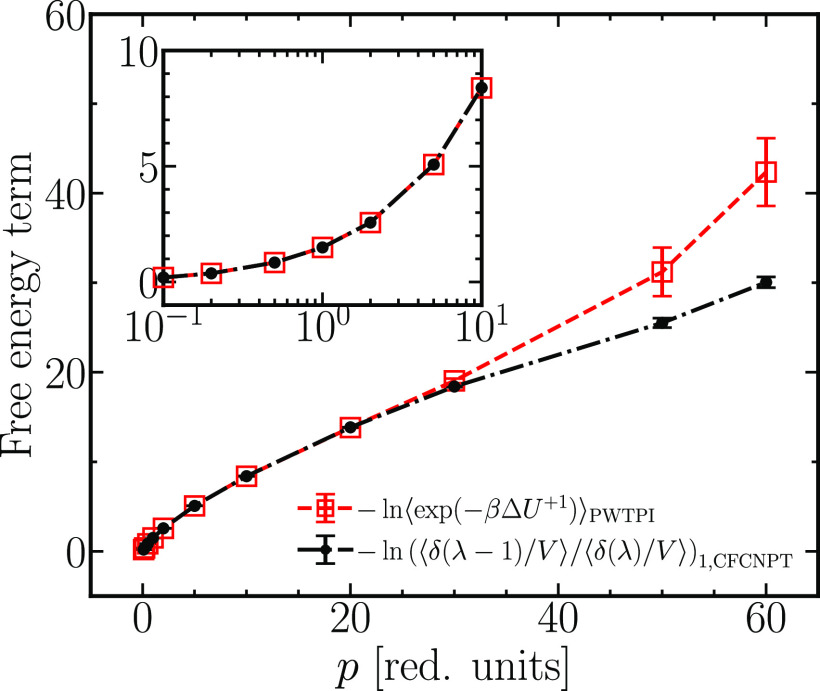
Comparison of the free energy terms computed by using the CFCMC and PWTPI methods for the insertion of a single WCA test molecule. The inset zooms in on the region *p* ≤ 10.

**Figure 11:**
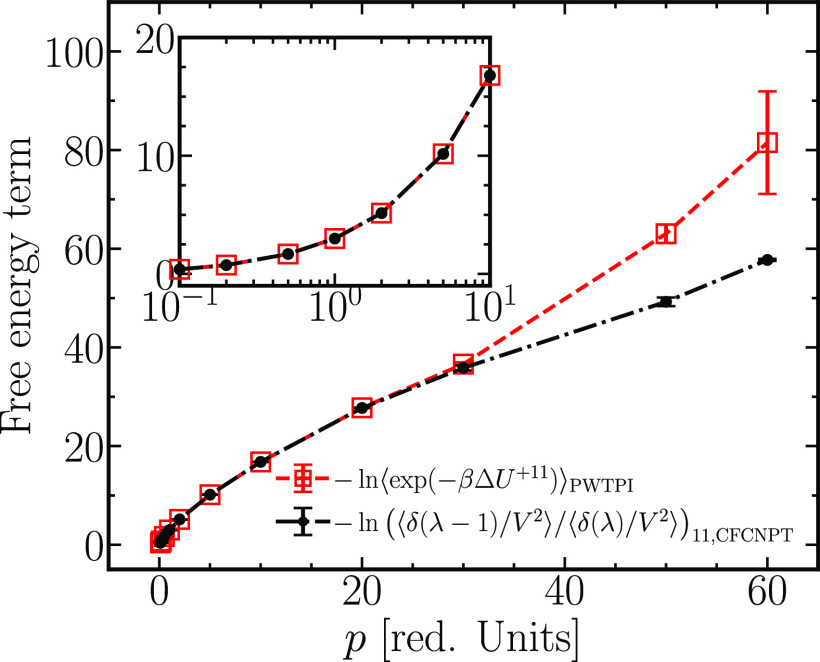
Comparison of the free energy terms computed using the CFCMC and PWTPI methods for the insertion of two WCA test molecules. The inset zooms in on the region *p* ≤ 10.

We show numerically that traditional test molecule insertion methods such as the WTPI and the PWTPI methods perform poorly in dense systems in comparison to the CFCMC method. Simulations are performed in the *NPT* and CFCNPT ensembles, where whole test molecules and fractional molecules are inserted, respectively, in a single-component system consisting of 100 WCA molecules. The temperature is fixed at 2 (reduced units), and the pressure is varied between 0.1 and 60 (reduced units), respectively. Fifteen independent simulations are performed at every pressure, and the mean and uncertainty of an observable are calculated using the method of block averages, as mentioned in Section 3. The densities computed from the PWTPI and CFCMC methods agree within 0.5% of each other, despite varying by a factor of 30 over the entire pressure range. From Figure 10, the free energy term for a single test molecule insertion from the PWTPI and the CFCMC methods agree excellently for *p* below 30. At higher pressures, the disagreement between the methods is evident, and there are notable uncertainties in the PWTPI method’s free energy predictions. An identical conclusion follows for the free energy terms related to the two-molecule insertions; see Figure 11. Large uncertainties and the overestimation of the insertion free energies are typical of single-step insertion methods such as the WTPI and PWTPI in dense systems.^10^ Our conclusions match those of Torres-Knoop et al.,^25^ who showed in the context of adsorption in porous materials that single-stage insertions yield unphysical results for thermodynamic quantities. The authors showed that the CFCMC technique outperforms the CBMC technique (another single-step insertion technique) in terms of insertion efficiency in dense systems.

## 5 Conclusions

We have introduced a new method to calculate thermodynamic factors for diffusion of binary and multicomponent systems, inspired by the Permuted Widom Test Particle Insertion (PWTPI) method.^21,22^ The PWTPI method, just like the conventional Widom’s Test Particle Insertion (WTPI) method, struggles with molecule insertions at large densities. Differentiation of activity coefficient models provides an indirect route to calculate thermodynamic factors for diffusion, but this route is unattractive since their prediction varies (significantly) with the choice of the model. The accuracy in the prediction of thermodynamic factors is also hampered by the quality of the fit to experimental vapor–liquid equilibrium (VLE) data. The CFCMC method uses groups of fractional molecules to facilitate molecule insertions and removals in stages. This feature of the CFCMC method alleviates the aforementioned deficiency of the PWTPI method at high densities. It also provides a direct route to accurately calculate the thermodynamic factors for diffusion in molecular systems from multiple simulations. Following Balaji et al.,^22^ we provide expressions for the thermodynamic factors for diffusion by eliminating the finite system size effects.

An equivalence was first established between the expressions for thermodynamic factors calculated from the CFCMC and PWTPI methods using the technique outlined by Poursaeidesfahani et al.^29^ The resulting expressions for the thermodynamic factors from the CFCMC method were benchmarked to the PWTPI method for a binary system consisting of Lennard-Jones molecules^21^ and a ternary system of WCA molecules.^22^ Excellent agreement was found for the binary and ternary systems between the two methods. The CFCMC method was then used to calculate the thermodynamic factors for binary mixtures of carbon dioxide and hydrogen at *p* = 50 bar and 500 bar and *T* = 323.15 K. The large liquid-like densities of binary mixtures with minute quantities of hydrogen (*x*_H_2__ ≈ 0.1) will pose significant challenges for the calculation of thermodynamic factors using the PWTPI method. We show that the thermodynamic factors calculated using the CFCMC method are in excellent agreement with corresponding values from the NIST REFPROP database.^42^ Our method demonstrates an efficient route to accurately predict thermodynamic factors in dense systems, even with relatively small system sizes consisting of approximately 100 molecules.
